# A pilot radiogenomic study of DIPG reveals distinct subgroups with unique clinical trajectories and therapeutic targets

**DOI:** 10.1186/s40478-020-01107-0

**Published:** 2021-01-11

**Authors:** Xiaoting Zhu, Margot A. Lazow, Austin Schafer, Allison Bartlett, Shiva Senthil Kumar, Deepak Kumar Mishra, Phillip Dexheimer, Mariko DeWire, Christine Fuller, James L. Leach, Maryam Fouladi, Rachid Drissi

**Affiliations:** 1 Brain Tumor Center, Division of Oncology, Cincinnati Children’s Hospital Medical Center, Cincinnati, OH USA; 2 Department of Electrical Engineering and Computer Science, University of Cincinnati College of Engineering and Applied Science, Cincinnati, OH USA; 3 The Ohio State University College of Medicine, Columbus, OH USA; 4 Center for Childhood Cancer & Blood Disorders, Nationwide Children’s Hospital, Columbus, OH USA; 5 Department of Biomedical Informatics, Cincinnati Children’s Hospital Medical Center, Cincinnati, OH USA; 6 Department of Pathology, Upstate Medical University, Syracuse, NY USA; 7 Department of Radiology and Medical Imaging, Cincinnati Children’s Hospital Medical Center, Cincinnati, OH USA; 8 Department of Radiology, University of Cincinnati College of Medicine, Cincinnati, OH USA; 9 Pediatric Neuro-Oncology Program, Nationwide Children’s Hospital, Columbus, OH USA

**Keywords:** Radiogenomics, DIPG, Serial MR imaging, Overall survival, Molecular subgrouping

## Abstract

An adequate understanding of the relationships between radiographic and genomic features in diffuse intrinsic pontine glioma (DIPG) is essential, especially in the absence of universal biopsy, to further characterize the molecular heterogeneity of this disease and determine which patients are most likely to respond to biologically-driven therapies. Here, a radiogenomics analytic approach was applied to a cohort of 28 patients with DIPG. Tumor size and imaging characteristics from all available serial MRIs were evaluated by a neuro-radiologist, and patients were divided into three radiographic response groups (partial response [PR], stable disease [SD], progressive disease [PD]) based on MRI within 2 months of radiotherapy (RT) completion. Whole genome and RNA sequencing were performed on autopsy tumor specimens. We report several key, therapeutically-relevant findings: (1) Certain radiologic features on first and subsequent post-RT MRIs are associated with worse overall survival, including PD following irradiation as well as present, new, and/or increasing peripheral ring enhancement, necrosis, and diffusion restriction. (2) Upregulation of EMT-related genes and distant tumor spread at autopsy are observed in a subset of DIPG patients who exhibit poorer radiographic response to irradiation and/or higher likelihood of harboring *H3F3A* mutations, suggesting possible benefit of upfront craniospinal irradiation. (3) Additional genetic aberrations were identified, including *DYNC1LI1* mutations in a subgroup of patients with PR on post-RT MRI; further investigation into potential roles in DIPG tumorigenesis and/or treatment sensitivity is necessary. (4) Whereas most DIPG tumors have an immunologically “cold” microenvironment, there appears to be a subset which harbor a more inflammatory genomic profile and/or higher mutational burden, with a trend toward improved overall survival and more favorable radiographic response to irradiation, in whom immunotherapy should be considered. This study has begun elucidating relationships between post-RT radiographic response with DIPG molecular profiles, revealing radiogenomically distinct subgroups with unique clinical trajectories and therapeutic targets.

## Introduction

Despite recent advances providing valuable insight into the underlying biology of diffuse intrinsic pontine glioma (DIPG), the most common brainstem tumor of childhood, prognosis remains dismal, with median overall survival less than 12 months [2, 30, 42]. DIPG is diagnosed based on characteristic radiographic and clinical features, and although there is emerging evidence supporting the safety and feasibility of diagnostic surgical biopsy, its role is still controversial [1, 8, 26, 63]. However, recent genome-wide sequencing analyses have been possible on DIPG tumor samples from biopsy and autopsy, revealing recurring driver mutations in the genes encoding histone H3.3 (*H3F3A*) or H3.1 (*HIST1H3B*), resulting in key substitution of lysine to methionine at position 27 (K27M) [66, 75] with subsequent aberrant transcription and associated prognostic significance [10, 40]. Additional somatic genetic alterations of receptor tyrosine kinases, cell cycle regulators, mediators of DNA repair, and/or PI3K/AKT/mTOR signaling have been identified in DIPG and may define unique molecular subgroups [7, 50, 56, 76]. While clinical trials over the past decades have failed to improve outcomes for patients with DIPG, growing knowledge of the genetic and epigenetic drivers of this disease may facilitate new targeted therapies as well as classification into biologically and clinically distinct subtypes [7, 71, 72]. Furthermore, given the relative resistance of DIPG to conventional therapies, a future multimodal treatment approach has been proposed, incorporating a combination of genetic/epigenetic, microenvironmental, and immunotherapeutic targets [2, 52]. In order to further characterize the molecular and clinical heterogeneity of this disease and determine which patients are most likely to respond to biologically-driven therapies, especially in the absence of universal diagnostic biopsy, an improved understanding of the relationships between radiographic and genomic characteristics in DIPG will be critically important.

Radiogenomics is a rapidly growing field within cancer research which integrates imaging phenotypes with tumor molecular profiles to build predictive models guiding diagnosis, treatment, and prognosis [4]. Most radiogenomics research to date has focused on elucidating key radiographic and genetic correlations in glioblastoma multiforme and other high-grade gliomas in adults [3, 20, 23, 27, 36, 38, 60, 74], which similar to DIPG, have an especially poor prognosis and a critical need for non-invasive assessment tools. Utilizing emerging innovative radiogenomic approaches, associations have been identified between prognostically relevant gene expression profiles, presence or absence of genetic alterations, and chromosomal gains/losses in glioblastoma with macroscopic magnetic resonance imaging (MRI) features, such as enhancement, necrosis, edema, tumor size, and/or measures of cerebral blood volume [3, 27, 36, 38, 60, 74]. Although previously limited to adult central nervous system (CNS) tumors, radiogenomic analyses have recently been expanded to pediatric neuro-oncology, with the discovery of predictive MRI correlates of molecular subgroups in medulloblastoma and atypical teratoid/rhabdoid tumors [16, 17, 35, 55, 58].

Among patients with DIPG, emerging research has demonstrated key radiologic features on diagnostic MRI suggestive of worse overall survival, including ring enhancement, extrapontine extension, larger tumor size, necrosis, distant metastatic disease, and/or lower apparent diffusion coefficient (ADC) values [33, 39, 45, 62]. Jaimes et al. recently identified significant differences in specific MRI characteristics at diagnosis (volume of enhancing tumor and ADC histogram parameters) among unique molecular subgroups of DIPG defined by the presence or absence of *MGMT*, *EGFR*, and *H3F3A* versus *HIST1H3B*/C mutations [37]. This aforementioned work importantly supports the feasibility of utilizing radiogenomics constructs to study DIPG, but has been limited thus far by incorporation of diagnostic imaging features only (i.e., lacking radiographic characteristics at subsequent time points following treatment) as well as the absence of more extensive genetic sequencing for correlative analysis. An enhanced understanding of how the detailed genomic profiles of DIPG tumors correlate with MR imaging phenotypes, both at diagnosis and serially over time, including in response to radiotherapy, is essential to guide treatment decisions and prognosis conversations.

Herein, we apply a radiogenomics analytic approach to a cohort of patients with DIPG in order to investigate potential relationships between MRI features, especially at post-radiation time points, with the underlying molecular landscape of these tumors, assessed via whole genome and RNA sequencing.

## Materials and methods

### Clinical cohort

This retrospective radiogenomics study was approved by the Institutional Review Board (IRB) at Cincinnati Children’s Hospital Medical Center (CCHMC; IRB ID: 2019–1220) and included patients enrolled on the CCHMC investigator-initiated Pediatric Brain Tumor Repository (PBTR), a multidisciplinary approach to pediatric brain tumor autopsy donation [19]. All but one patient were concurrently enrolled on the International DIPG Registry. All tumor specimens were collected after written informed consent was obtained from patients and families in accordance with approved IRB studies. DIPG tumor specimens with their matched normal brain tissue (right or left frontal lobe) were obtained from autopsy (CCHMC-PBTR cohort) as described below. Clinical data, including age, sex, presenting symptoms, treatment details, and overall survival (defined as the time from diagnosis to death), were abstracted from the patients’ electronic health records and subsequently de-identified.

### Autopsy protocol

The median post-mortem interval (PMI, time from death to autopsy evaluation) was 13 h (range 4–30 h). DIPG tumor samples were selected for analysis by an experienced neuro-pathologist (CF) from pontine areas with grossly evidence disease that correlated with contrast enhancement on imaging (when applicable), as previously described [32]. The frontal lobe was chosen for normal brain comparison to avoid tumor contamination, given its maximal distance from the brainstem. In all cases, frontal lobe tissue was included for analysis only after ensuring no findings to suggest tumor involvement by gross inspection and on corresponding MRI (i.e., confirming absence of T2 hyperintensity or contrast enhancement). Furthermore, all samples submitted for genomic analysis first underwent inspection by frozen section to determine whether they represented tumor or normal; additionally, tissue was selected from immediately adjacent to these regions and examined by routine histology following formalin fixation to ensure concordance with frozen section findings. If tumor cells were suspected by histologic evaluation in areas sampled as normal frontal lobe tissue, H3 K27M-mutant immunohistochemistry was performed to ensure no malignant cells were present. Finally, Sanger sequencing performed on all normal frontal lobe tissue included in the analysis below also confirmed the absence of H3 K27M mutations.

### Radiographic evaluation

MRIs for all patients included in this study were centrally reviewed by an American Board of Radiology-certified, fellowship-trained pediatric neuro-radiologist who has a certificate of added qualification in neuroradiology, extensive experience evaluating neuro-imaging in clinical trials of patients with DIPG, and is a central reviewer for the International DIPG Registry (JL). After appropriate patient de-identification, baseline imaging was assessed in a standardized fashion (blinded to outcome or genomic data) and included assessment of bi-dimensional tumor measurements (anteroposterior [AP]) x transverse [TR]), necrosis, peripheral enhancement, extrapontine extension, and diffusion restriction (amongst other features), as previously described [45]. Regions of necrosis were defined as areas of well-defined, non-enhancing, fluid-like signal present within the tumor, with marked hypoperfusion on perfusion imaging if performed; most exhibited peripheral, rim-like enhancement when contrast was administered. The extent of necrosis, enhancement, and diffusion restriction were assessed visually at each time point, compared with the previous time point, and were delineated as stable, increased, or decreased. Bi-dimensional measurements of enhancement and necrosis were also performed (when present) and utilized to confirm the aforementioned subjective changes when applicable. The product of AP and TR dimensions of necrotic and enhancing regions were calculated and summed if more than one enhancing or necrotic region existed in the tumor. All available MRIs for each patient were reviewed, with a focus on imaging at diagnosis, first post-radiotherapy (post-RT) assessment, best response, and first radiographic progression (encompassing 87 MRI exams in total). The first post-RT assessment occurred during the time period between 10 days and 2 months of completion of irradiation; if more than one MRI was obtained during this window, the imaging closest to 1 month post-RT completion was selected. Best response was defined as the smallest tumor size reached after diagnosis. First radiographic progression was defined as the first instance of a ≥ 25% increase in tumor size compared to best response or the largest increase in size post-best response if a 25% increase was never reached.

### Histone mutation assessment

H3 K27M-mutant-specific immunohistochemistry was performed on tumor samples. Histone point mutation status (*H3F3A* [H3.3] and *HIST1H3B* [H3.1]) was further validated by Sanger sequencing.

### RNA sequencing

RNA extraction was carried out using the RNeasy Plus Mini Kit (Qiagen) according to manufacturer’s instructions and quantified using the Qubit RNA BR assay kit (Invitrogen). The TruSeq RNA Access kit was used for RNA library preparation. Approximately 60 M paired-ended reads were generated for each sample. Pseudoalignment and quantification were performed by Kallisto 0.44.0 [6] against the human transcriptome build GRCh38. The expression quantifications, normalization, and differential expression analyses were conducted using the Bioconductor package DESeq2 [49] and converted to normalized gene counts. Relative gene expression was represented by log2(x + 1). The Wald-test was used to evaluate differential expression. Significance of differential expression was defined as absolute log_2_ fold change > 1 and adjusted *p* value < 0.05. Gene set overrepresentation and gene set enrichment analysis were conducted using Gene Ontology KEGG pathways and MSigDB [46].

### Whole genome sequencing

DNA extraction was carried out using the Gentra Puregene Tissue Kit (Qiagen) according to manufacturer’s instructions and quantified using Qubit dsDNA HS assay kit (Invitrogen). The TruSeq Nano DNA library kit was used for whole genome sequencing library preparation. An average depth of approximately 30 × was obtained for tumor and normal tissue. Data was processed using the platform VIVA-v2.1.0 (https://viva.research.cchmc.org). Variants that passed the quality filters were further selected based on expected moderate or high impact on encoding proteins; variants in coding regions (exons) were selected for functional analysis.

### Statistical analysis

Continuous and categorical variables were described by median (range) and frequency (percent), respectively. The Wilcoxon Rank-Sum test and Kruskal–Wallis tests were used to compare continuous variables among post-RT response groups. Correlations between continuous-scale variables were calculated using the Spearman correlation coefficient. The Fisher exact test was used to compare categorical variables variable among groups. The Log-rank test and Univariate Cox proportional hazard test was used to evaluate for clinical, radiographic and genomic predictors of overall survival. *p* values < 0.05 were considered statistically significant. For RNA sequencing-related outcomes and correlations analyzed, the false discovery rate (FDR) was calculated using the Benjamini–Hochberg method to account for multiple hypothesis tests. Tests with a calculated FDR of 10% were considered statistically significant after FDR adjustment. Corrections for multiple testing for additional analyses did not yield statistically significant results, but original *p* values were reported to offer trends that deserve corroboration in future, larger-scale study planned and as such, should be interpreted cautiously.

## Results

### Patient cohort and clinical characteristics

Twenty-eight patients with DIPG were included in this study, with clinical characteristics and treatment received summarized in Table 1. Median age at diagnosis was 7.3 years (Range 1.8–24.0) and median overall survival was 11.5 months (Range 0–82). Twenty-six (93%) patients received radiotherapy (photon irradiation [n = 24]; proton irradiation [n = 2]), and five (18%) patients underwent re-irradiation (median time of re-irradiation from completion of initial radiotherapy: 12 months (Range 5–61), all with photon). Twenty-five (89%) patients received systemic therapy between diagnosis and death, including a radiosensitizing agent during RT in 11 (44%) patients and/or adjuvant therapy in 22 (79%) patients. One patient had a non-contiguous presumed cerebellar metastatic lesion at diagnosis; there was no evidence of distant metastatic disease at diagnosis in the remaining patients, including in nine patients who had diagnostic spine MRI performed (all negative). Distant and/or metastatic disease at autopsy was observed in nine patients (32%).

**Table 1:** Clinical Characteristics of the 28 patients in this cohort

Characteristics	N	%
**Age (years)**		
Median: 7.3 years (range 2.3–24)		
< 3	2	7
3–10	16	57
10 +	10	36
**Gender**		
Female	15	54
Male	13	46
**Overall survival (months)**		
Median: 11.5 months (range 0–82)		
< 12	14	50
12–24	11	39
> 24	3	11
**Symptom duration (months)**		
< 6	19	68
6–12	2	7
12–24	5	18
Not available	2	7
**Radiotherapy**	26	93
Photon irradiation	24	86
Proton irradiation	2	7
**Re-irradiation**	5	18
**Systemc therapy**	25	89
Radiosensitizer	11	44
Adjuvant therapy	22	79
**Specific systemic therapy agent(s) received**		
Bevacizumab	8	30
Vorinostat (HDAC inhibitor)	7	26
CDK4/6 inhibitor (ribcoclib [n = 6], palbociclib [n = 1])	7	26
Everolimus (mTOR inhibitor)	4	15
Irinotecan	4	15
Temozolomide	3	11
PARP inhibitor	3	11
Tyrosine kinase inhibitor (cabozantinib [n = 1], pazopanib [n = 1], dasatinib [n = 1])	3	11
WEE-1 inhibitor	2	7
Tivantinib (cMET inhibitor)	2	7
ONC201	1	4
Trametinib (MEK inhibitor)	1	4
Imetelstat (Telomerase inhibitor)	1	4
**Distant metastases at diagnosis**^a^	1	4
**Distant metastases at autopsy**	9	32
Cerebral cortex	7	25
Cerebellar peduncles and/or cerebellum	4	14
Thalamus and/or subthalamus	3	11
Basal ganglia	3	11
Cervical spinal cord	1	4
**Histone mutation status**		
H3.3	20	71
H3.1	7	25
WT	1	4
**Extent of pathological spread (autopsy)**		
Extension beyond brainstem	22	79
Limited to brainstem	5	18
Insuffient information	1	4
**Location of pathologic spread (autopsy)**		
Cerebellar peduncles and/or cerebellum	21	75
Thalamus and/or subthalamus	15	54
Cervical cord	13	46
Basal ganglia	8	29
Cortex	2	7

^a^Assessed by MRI at diagnosis. Spine MRI was obtained in 9 patients at diagnosis, all without evidence of spinal metastatic disease

### Presence of genomic alterations and overall survival

#### Known clinically relevant genomic alterations in DIPG

**Fig. 1:**
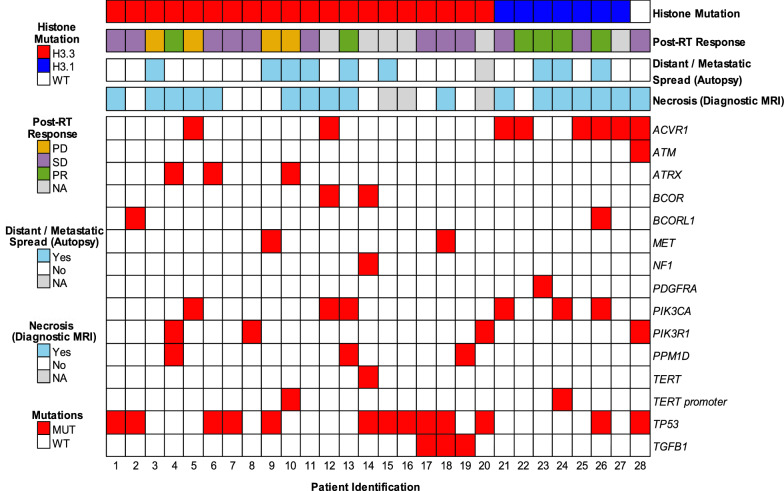
Individual genomic profiles of the DIPG tumors in our cohort with corresponding key clinical information. Each patient is represented by a vertical column, with respective color-coding (defined by the key) indicating histone mutation status (H3.3, H3.1, or wild-type [WT]), radiographic response on first post-RT MRI (*PR* partial response; *SD* stable disease; *PD* progressive disease and NA, if lacking imaging data), presence of distant tumor spread and/or metastatic disease at autopsy, presence of necrosis on diagnostic MRI, and presence of mutations in genes with known clinical relevance to DIPG

Sanger sequencing of tumor autopsy specimens was performed for all 28 patients and revealed *H3F3A* (H3.3) mutations in 20 (71%) patients and *HIST1H3B* (H3.1) mutations in seven (25%) patients; only one patient had wild-type genomic results at these histone loci (Fig. 1). There was 100% concordance between H3 K27M-mutant specific immunohistochemistry and Sanger sequencing for histone mutation identification. There was no significant difference in overall survival identified between patients whose tumors harbored H3.3 and H3.1 mutations (median: 11 vs 15 months; *p* = 0.78). Additional clinically relevant genetic alterations commonly identified in our patient cohort included aberrations in *TP53* (n = 13 [46%]), *ACRV1* (n = 8 [29%]), *PIK3A* (n = 6 [21%]), *PIK3R1* (n = 4 [14%]), *ATRX* (n = 3 [11%]), *PPM1D* (n = 3 [11%]), *MET* (n = 2 [7%]), *BCOR* (n = 2 [7%]), and *BCORL1* (n = 2 [7%]) (Fig. 1). There was a trend toward higher proportion of *TP53* mutations in *H3F3A-* versus *HIST1H3B*-mutant tumors (55% vs 14%; *p* = 0.084), and a significantly higher proportion of *ACVR1* mutations in tumors with *HIST1H3B* compared to *H3F3A* mutations (71% vs 10%; *p* = 0.0047). Patients whose tumors harbored *MET* alterations had poorer overall survival compared to patients with *MET*-wild type tumors (median: 7 vs 12 months; hazard ratio [HR] 6.2, *p* = 0.031). Presence or absence of the remaining aforementioned genetic alterations were not found to independently predict overall survival (Table 2).

**Table 2:** Prevalence and prognostic significance of clinically relevant gene mutations in the cohort (n = 28)

Gene	N (%)	Median OS (months)			
		Mutant	Wild-type	Hazard ratio	*p* value
*H3F3A*	20 (71%)	11	15	1.00	1.00
*HIST1H3B*	7 (25%)	15	11	1.20	0.69
*TP53*	13 (46%)	12	11	0.71	0.39
*ACVR1*	8 (29%)	11.5	11.5	1.55	0.32
*PDGFRA*	1 (4%)	24	11	0.45	0.43
*ATM*	1 (4%)	19	11	0.52	0.52
*PIK3CA*	6 (21%)	11.5	11.5	1.46	0.45
*PIK3R1*	4 (14%)	14.5	11.5	0.85	0.77
*ARTX*	3 (11%)	11	12	1.40	0.59
*PPM1D*	3 (11%)	11	12	0.81	0.74
*BCOR*	2 (7%)	46	11.5	0.25	0.19
*BCORL1*	2 (7%)	23.5	11	0.45	0.29
*MET*	2 (7%)	7	12	6.20	0.03
*OR7E24*	3 (11%)	32	11	0.11	0.04
*SRGAP3*	5 (18%)	10	12	2.90	0.05
*HLA-B*	3 (11%)	32	11	0.14	0.06
*HLA-C*	3 (11%)	32	11	0.13	0.05

#### New genomic findings

Sequencing revealed additional likely pathogenic mutations in other genes, not previously described in DIPG to our knowledge, which impacted overall survival. Patients whose tumors harbored alterations in *SRGAP3* (n = 5 [18%], all with mutations resulting in an identical amino acid change from valine to alanine at position 1081; Fig. 1) had poorer overall survival compared to patients with *SRGAP3*-wild type tumors (median: 10 vs 12 months; HR 2.9, *p* = 0.047), whereas mutations in *OR7E24* (n = 3 [11%]) were associated with improved overall survival (median: 32 vs 11 months; HR 0.11, *p* = 0.036). Trends toward improved overall survival were also seen in patients whose tumors harbored alterations in human leukocyte antigen (HLA) genes *HLA-B* (n = 3 [11%]; median: 32 vs 11 months; HR 0.14, *p* = 0.057) and *HLA-C* (n = 3 [11%]; median: 32 vs 11 months, HR 0.13, *p* = 0.053); note: *HLA-B* and *HLA-C* mutations were found in four total patients (Fig. 1); the tumors of two patients’ harbored co-occurring *HLA-B* and *HLA-C* mutations (Patient# 2 and 14, whose tumors were also characterized by *OR7E24* mutations); two additional patients’ tumors had single mutations in *HLA-B* (Patient# 18) or *HLA-C* (Patient# 20).

### Differentially expressed genes between paired tumor and normal brain tissue

**Fig. 2:**
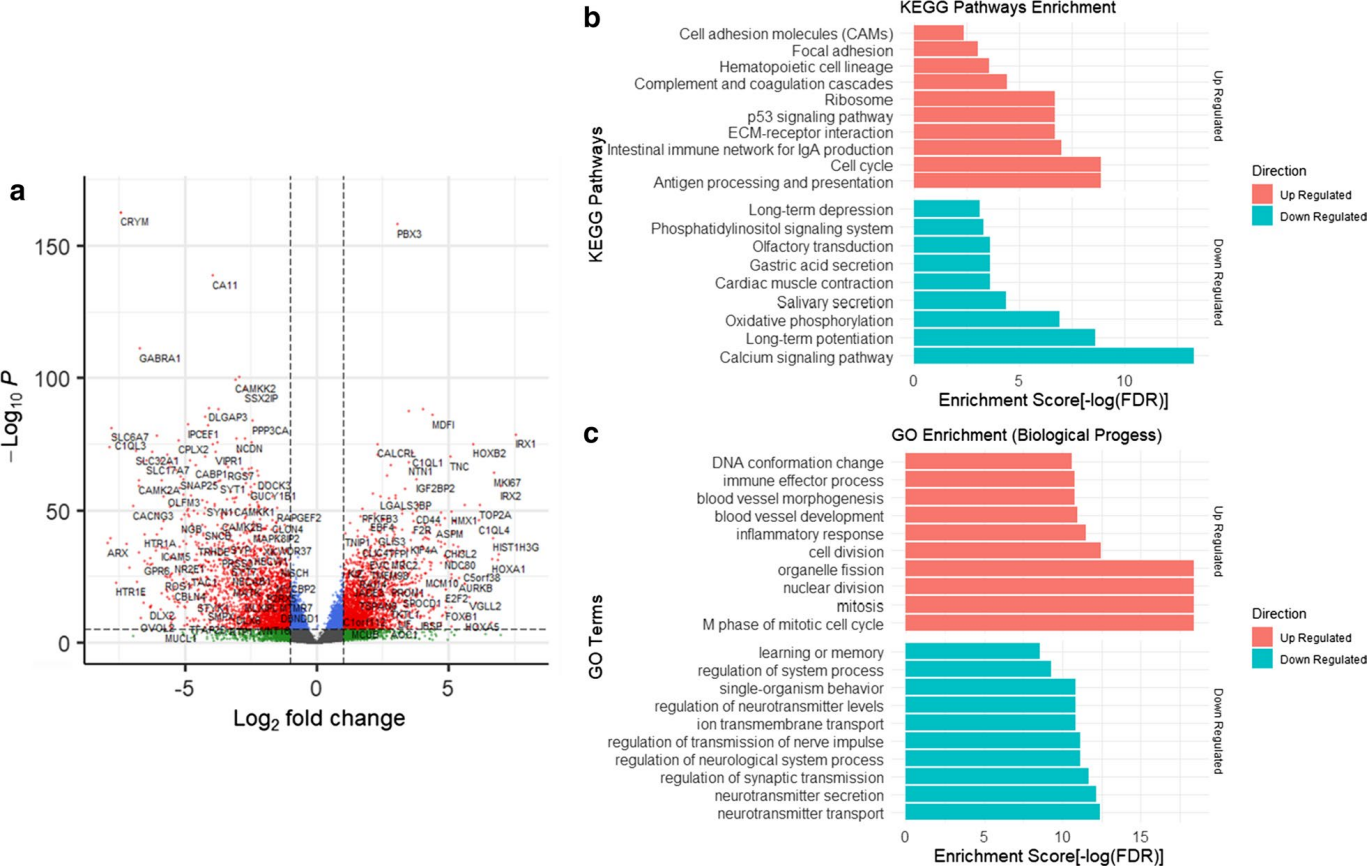
Differential gene expression between tumor and normal brain tissue. **a** Differentially expressed genes (DEGs) between DIPG tumor and normal brain (frontal lobe) in biologically relevant pathways are plotted, with adjusted *p* value < 0.05 and |log_2_ Fold Change|> 1. **b** KEGG pathways associated with the DEGs, with false discovery rate (FDR). **c** Gene ontology (GO) biological processes associated with the DEGs with FDR. For **b**, **c** upregulated pathways are illustrated in red and downregulated pathways are illustrated in green

Paired post-mortem tumor and normal brain (right or left frontal lobe) tissue samples were available for all 28 patients. RNA sequencing was performed on all paired tissue samples to reveal transcriptomic variations and identify differentially expressed genes. Comparing tumor to corresponding healthy brain tissue, there was significant upregulation of genes within the extracellular-matrix receptor interaction, epithelial-mesenchymal transition, p53 signaling, and cell cycle regulation functional pathways, and significant downregulation of genes within the oxidative phosphorylation and calcium signaling pathways (Fig. 2). Furthermore, analysis of bulk RNA sequencing data revealed consistently higher expression of specific effector genes involved in extracellular-matrix receptor interaction (*COL1A*, *FN1*) and epithelial-mesenchymal transition (*VIM*, *MMP2*) in all tumors compared to normal brain tissue (these results were validated in a subset of patients via real-time PCR [data not shown]). Finally, enrichment of epithelial-mesenchymal transition-related genes was observed in patients who did not undergo re-irradiation (n = 23) compared with patients who received re-irradiation (n = 5).

### Imaging features and overall survival

#### Diagnostic radiographic characteristics

Diagnostic MRI was available for review for 25 of 28 patients. Imaging features at diagnosis that correlated with poorer overall survival included presence of peripheral ring enhancement (HR 4.2, *p* = 0.008) and necrosis (HR 3.1, *p* = 0.029) (Table 3). A summary of all diagnostic imaging data is included in Additional file 1: Table S1. No association between steroid use at the time of diagnostic MRI and the presence or size of relevant radiographic features was observed.

**Table 3:** Prevalence and prognostic significance of MR imaging features at diagnosis, first post-RT assessment, best response, and first radiographic progression

	Diagnosis (N = 25)			First post-RT assessment (N = 22)			Best response (N = 22)			First radiographic progression (N = 18)		
	N (%)	Hazard ratio	*p* value	N (%)	Hazard ratio	*p* value	N (%)	Hazard ratio	*p* value	N (%)	Hazard ratio	*p* value
**Necrosis**	17 (68%)	**3.1**	**0.029**	16 (73%)	**6.3**	**0.0049**	17 (77%)	**7.9**	**0.0076**	12 (67%)	**5.6**	**0.0099**
New	–	–	–	8 (36%)	**3.3**	**0.025**	5 (23%)	2.4	0.12	7 (39%)	**3.2**	**0.03**
Increasing	–	–	–	12 (55%)	**3.6**	**0.01**	6 (27%)	3.1	**0.033**	8 (44%)	**25**	**0.003**
New and/or increasing	–	–	–	12 (55%)	**3.6**	**0.01**	6 (27%)	3.1	**0.033**	9 (50%)	**4.3**	**0.0078**
Decreasing	–	–	–	4 (18%)	1.4	0.53	10 (45%)	1.9	0.16	3 (17%)	0.94	0.93
**Enhancement**	21 (84%)	1.9	0.27	17 (81%)	3.5	0.05	18 (86%)	4	0.071	14 (78%)	3.2	0.072
New	–	–	–	6 (29%)	2.6	0.075	4 (19%)	1.8	0.33	9 (50%)	**4.1**	**0.012**
Increasing	–	–	–	12 (57%)	**4.1**	**0.0066**	6 (29%)	1.6	0.33	9 (50%)	> > 1	1
New and/or increasing	–	–	–	12 (57%)	**4.1**	**0.0066**	6 (29%)	1.6	0.33	10 (56%)	**5.4**	**0.0036**
Decreasing	–	–	–	6 (29%)	0.64	0.37	11 (52%)	1.3	0.51	6 (33%)	0.49	0.18
Peripheral ring like	16 (64%)	**4.2**	**0.0077**	14 (67%)	**5.3**	**0.0045**	13 (62%)	**4.5**	**0.0059**	10 (56%)	**5.4**	**0.0036**
Patchy ill defined	14 (56%)	0.96	0.93	11 (52%)	1.6	0.32	14 (67%)	1.3	0.57	12 (67%)	2.6	0.084
**Diffusion restriction**	15 (60%)	0.53	0.15	11 (50%)	**3.1**	**0.028**	11 (50%)	**3.1**	**0.028**	10 (56%)	1.3	0.59
New	–	–	–	7 (32%)	2.4	0.074	3 (14%)	**5.2**	**0.027**	6 (33%)	0.71	0.56
Increasing	–	–	–	7 (32%)	2.4	0.074	5 (23%)	**6.3**	**0.0054**	6 (33%)	0.71	0.56
New and/or increasing	–	–	–	7 (32%)	2.4	0.074	5 (23%)	**6.3**	**0.0054**	6 (33%)	0.71	0.56
Decreasing	–	–	–	6 (27%)	1.1	0.78	5 (23%)	0.73	0.55	2 (11%)	1.6	0.54
**Metastatic disease**	1 (4%)	5.5	0.13	2 (9%)	**11**	**0.017**	3 (14%)	1.2	0.75	4 (22%)	2	0.24
**Hemorrhage**	11 (44%)	1.3	0.56	16 (73%)	2.2	0.14	16 (73%)	2.2	0.14	13 (72%)	0.96	0.94
New	–	–	–	10 (45%)	1.9	0.14	9 (41%)	1.5	0.4	5 (28%)	0.69	0.52
Increasing	–	–	–	9 (41%)	1.9	0.14	8 (36%)	1.5	0.42	4 (22%)	0.8	0.74
New and/or increasing	–	–	–	10 (45%)	1.9	0.14	9 (41%)	1.5	0.4	5 (28%)	0.69	0.52
Decreasing	–	–	–	4 (18%)	0.99	0.99	3 (14%)	1.8	0.38	1 (6%)	0.81	0.84

#### Serial imaging

**Fig. 3:**
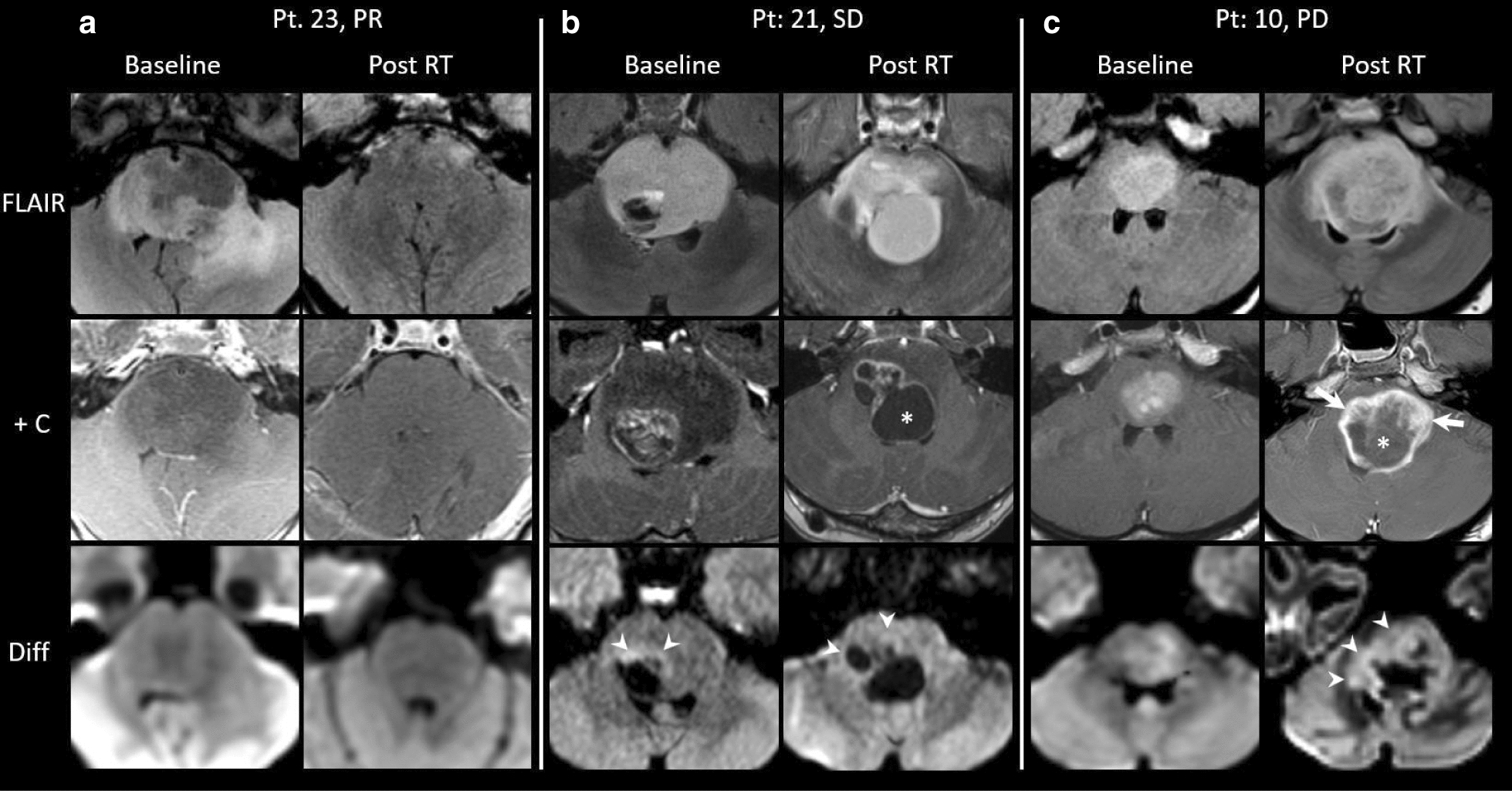
Representative T2 FLAIR, Post-contrast T1 (+ C), and Diffusion-weighted (diff) sequences of diagnostic and first post-radiotherapy (post-RT) MRIs from patients within the three radiographic response groups. **a** Imaging from patient #23 (overall survival [OS]: 24 months) whose tumor exhibited a partial response (PR) on first post-RT MRI, with no associated contrast enhancement, necrosis, or diffusion restriction. **b** Imaging from patient #21 (OS: 11 months) whose tumor exhibited stable disease (SD) on first post-RT MRI, with increasing necrosis (*) and enhancement, but similar diffusion restriction along enhancing regions (arrowheads). **c** Imaging from patient #10 (OS: 5 months) whose tumor exhibited progressive disease (PD) on first post-RT MRI, with associated increased necrosis (*), enhancement (arrows), and diffusion restriction (arrowheads). Areas of diffusion restriction were confirmed on apparent diffusivity maps

First post-RT MRI data were available for 22 patients (Fig. 3); median time from completion of irradiation to imaging was 27 days (Range 11–44 days). At least one additional MRI at subsequent clinical time points was available for 18 patients. In a univariate analysis using the Cox proportional-hazards model, the following radiographic characteristics on first post-RT MRI correlated with poorer overall survival: presence of peripheral ring enhancement (HR 5.3, *p* = 0.0045), new and/or increased peripheral ring enhancement from diagnostic MRI (HR 4.1, *p* = 0.0066), presence of necrosis (HR 6.3, *p* = 0.0049), new and/or increased necrosis from diagnostic MRI (HR 3.6, *p* = 0.01), and presence of diffusion restriction (HR 3.1, *p* = 0.028) (Table 3). A trend toward association between steroid use at the time of first post-RT MRI and size of necrosis at this timepoint was observed (*p* = 0.060); no additional correlations between concurrent steroid use were identified. On MRI obtained at the time of best response (which occurred at a median of 3.7 months [range 0.5–9.8 months] from completion of irradiation and coincided with the first post-RT MRI in 5 [23%] patients), the presence of peripheral ring enhancement (HR 4.5, *p* = 0.0059), new and/or increased necrosis from last MRI (HR 3.1, *p* = 0.03), and new and/or increased diffusion restriction (HR 6.3, *p* = 0.0054) were associated with worse overall survival. On MRI obtained at the time of first radiographic progression, correlations between the following imaging features and shorter overall survival were demonstrated: presence of peripheral ring enhancement (HR 5.4, *p* = 0.0036), new and/or increased enhancement from last MRI (HR 5.4, *p* = 0.0036), presence of necrosis (HR 5.6, *p* = 0.0099), and new and/or increased necrosis from last MRI (HR 4.3, *p* = 0.0078) (Table 3). Presence or change in diffusion restriction on this MRI was not associated with overall survival. Additionally, larger quantitative bi-dimensional measures of both necrosis and enhancement (dichotomized compared to the cohort median) demonstrated significant correlation with worse overall survival on all post-RT MRIs (first post-RT assessment, best response, and first progression; log-rank analyses: *p* < 0.05 for all).

### Correlation between post-RT MRI response and clinical and genomic characteristics

#### Classification into post-RT subgroups and clinical correlates

**Fig. 4:**
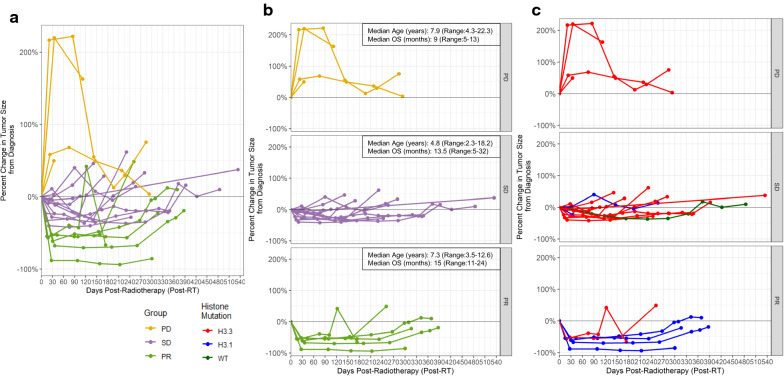
Radiographic tumor size change over time, with subclassification based on first post-RT MRI response. **a**, **b** Tumor size change across serial MRIs is demonstrated for all patients in the cohort with available imaging data (n = 22). Each dot represents a unique MRI time point, illustrating percent change in bi-dimensional (AP × TR) tumor size from baseline (diagnostic) MRI (y-axis) versus days post-completion of radiotherapy (x-axis). Individual patients’ imaging courses over time are illustrated by lines connecting these dots, which are color-coded based on radiographic response on first post-RT MRI (PD [yellow], SD [purple], PR [green]) **c** Distribution of histone mutation status (H3.3 [red], H3.1 [blue], or WT [dark green]) across the three post-RT imaging response groups

Using established criteria for radiographic disease evaluation from the Response Assessment in Neuro-Oncology (RANO) and Response Assessment in Pediatric Neuro-Oncology (RAPNO) working groups [13, 14], patients were classified into three groups based on first post-RT MRI, including (1) *progressive disease (PD)*: ≥ 25% increase in bi-dimensional (AP x TR) tumor measurements compared to diagnostic baseline, without subsequent decrease to pre-irradiation tumor dimensions or ability to wean off steroids within 6 months of irradiation completion (i.e., no evidence to suggest pseudoprogression) (n = 4), (2) *partial response (PR)*: ≥ 50% decrease in bi-dimensional tumor measurements (n = 6), or (3) *stable disease (SD)*: percent change in tumor size between 50% decrease and 25% increase of diagnostic baseline (i.e., neither progressive disease or partial response; n = 12) (Figs. 3 and 4). No significant differences in overall survival (*p* = 0.315) were identified when comparing these three groups separately (PD vs SD vs PR); however, patients with PD on post-RT MRI were found to have poorer overall survival compared with those without PD (i.e., SD or PR) (median: 9 vs 15 months; *p* = 0.039). There were no significant differences in the three groups with regard to age at diagnosis (*p* = 0.544) or the proportion of patients who received radiosensitizer therapy (*p* = 0.33), though the proportion of patients receiving concurrent steroids differed between response groups (PD > SD > PR; *p* = 0.01).

#### Association with other imaging features

A significant difference in the proportion of patients with new and/or increasing enhancement on first post-RT imaging was identified when comparing the three disease response groups (*p* = 0.032; PD [100%] > SD [64%] > PR [17%]; Fig. 3). No significant differences in the proportion of patients in the three disease response groups whose tumors exhibited necrosis, diffusion restriction, or peripheral ring enhancement specifically on diagnostic MRI or on first post-RT imaging were observed.

#### Histone 3.3 versus 3.1 alterations

All four (100%) patients with evidence of PD on post-RT MRI had somatic *H3F3A* (H3.3) mutations, whereas four of six (67%) patients with PR had somatic *HIST1H3B* (H3.1) mutations (Fig. 4c). When comparing the three post-RT response groups individually, there was a trend toward statistical difference in the proportion of *HIST1H3B* (H3.1) mutations (PR > SD > PD; *p* = 0.062). Furthermore, there was a significantly higher proportion of tumors harboring *HIST1H3B* (H3.1) mutations among patients with a PR on post-RT MRI versus patients without a PR (PD or SD) (*p* = 0.025).

#### Other genetic alterations

There were no significant differences in the proportion of patients whose tumors harbored known clinically relevant alterations of *TP53*, *ACRV1*, *PIK3CA*, *PIK3R1*, *ATRX*, *PPM1D*, *MET*, *BCOR*, or *BCORL1* among the three post-RT response groups. A trend toward a higher proportion of *PIK3CA* mutations was observed in patients who exhibited PR to irradiation compared to those without PR (PD or SD) [50% vs 13%, *p* = 0.10]. When the presence of additional genetic alterations detected on whole genome sequencing was compared among the three post-RT response groups, we identified a significant difference in the proportion of tumors harboring mutations in *DYNC1LI1* (gene encoding dynein cytoplasmic 1 light intermediate chain 1, a protein complex involved in conversion of energy from ATP hydrolysis into mitotic spindle function [59, 68]). *DYNC1LI1* mutations were detected in three of six (50%) of patients with PR post-RT, but in none of the patients with SD or PD (*p* = 0.012).

#### Comparison of differentially expressed genes between imaging response groups

**Fig. 5:**
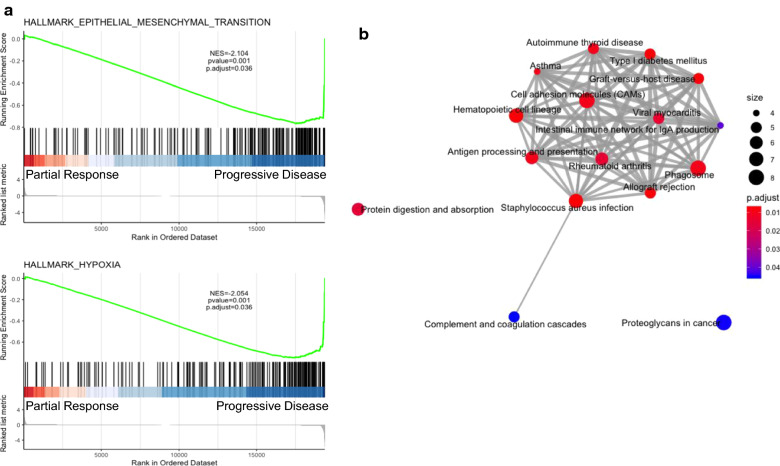
Genomic comparison between PD and PR post-RT response groups, based on Gene Set Enrichment Analysis (GSEA) and GO term enrichment analysis. **a** GSEA results, in which the total height of the curve reflects the extent of gene enrichment within given biological pathways, with corresponding normalized enrichment score (NES) and false discovery rate (FDR) reported. Upregulated genes in epithelial-mesenchymal transition and hypoxia pathways are enriched in the PD compared to PR post-RT response groups. **b** GO term enrichment analysis results, illustrating differential expression of genes associated with inflammation and immune-mediated pathways between the PD and PR post-RT response groups. The size of dot reflects the number of genes identified in each gene set

Gene set enrichment analysis (GSEA) was conducted to identify sets of genes overexpressed in specific post-RT disease response subgroups. Enrichment of genes associated with epithelial-mesenchymal transition was identified in patients with PD compared to those with either PR or SD on post-RT MRI; enrichment of hypoxia-associated genes was also observed in PD versus PR tumors (Fig. 5a). Additionally, when gene ontology KEGG pathway enrichment analyses were used to compare patients with PD and PR, there was significant differential gene expression in inflammation and immune-mediated pathways (i.e., antigen processing and presentation, cell adhesion molecules, allograft rejection, graft-versus-host disease, staphylococcus aureus infection, type I diabetes mellitus, autoimmune thyroid disease, asthma, phagosome, viral myocarditis, rheumatoid arthritis, proteoglycans in cancer, complement pathways, *p* < 0.05 for all; Fig. 5b). In order to ensure the aforementioned associations between differential gene expression and post-RT radiographic response were not confounded by histone mutational status, these analyses were also conducted comparing only *H3F3A*-mutant tumors within the PD and PR groups (n = 4 and 2, respectively), with the same findings. Lastly, a subanalysis of patients within the SD response group demonstrated enrichment of inflammatory pathways in the 50% of patients with longer periods of disease stability on subsequent MRIs, compared to the remaining 50% of patients with more rapid radiographic progression.

#### Metastatic disease and distant tumor spread at autopsy

The extent and location of metastatic disease/ distant tumor spread identified at autopsy (by histologic confirmation as well as correlation with post-mortem MRI [when available and applicable]) varied among response groups and in association with histone mutation status. Patients with PD post-RT were significantly more likely to have distant (cortical) metastases (75%) compared to those without PD (17%; *p* = 0.018). No significant difference was observed between PD and non-PD response groups for proportion with distant tumor spread involving the thalamus/subthalamus or cerebellum. In addition, patients whose tumors harbored *H3F3A* mutation were significantly more likely to have distant tumor spread to the cervical spinal cord on autopsy (62%) than *HIST1H3B*-mutant tumors (14%; *p* = 0.033). Presence of mutations in *ACVR1*, *PI3KCA*, and/or *PI3KR1* did not correlate with metastatic or distant disease. No significant correlation was seen between expression of the aforementioned subset of analyzed effector genes involved in extracellular-matrix receptor interaction (*COL1A*, *FN1*) or epithelial-mesenchymal transition (*VIM*, *MMP2*) and presence of distant metastatic/distant disease at autopsy (*p* > 0.05 for all). Finally, neither receipt of systemic therapy nor type of systemic therapy correlated with development of metastatic or distant disease.

### Immunological profiling

#### Immune subtypes and clinical correlates

**Fig. 6:**
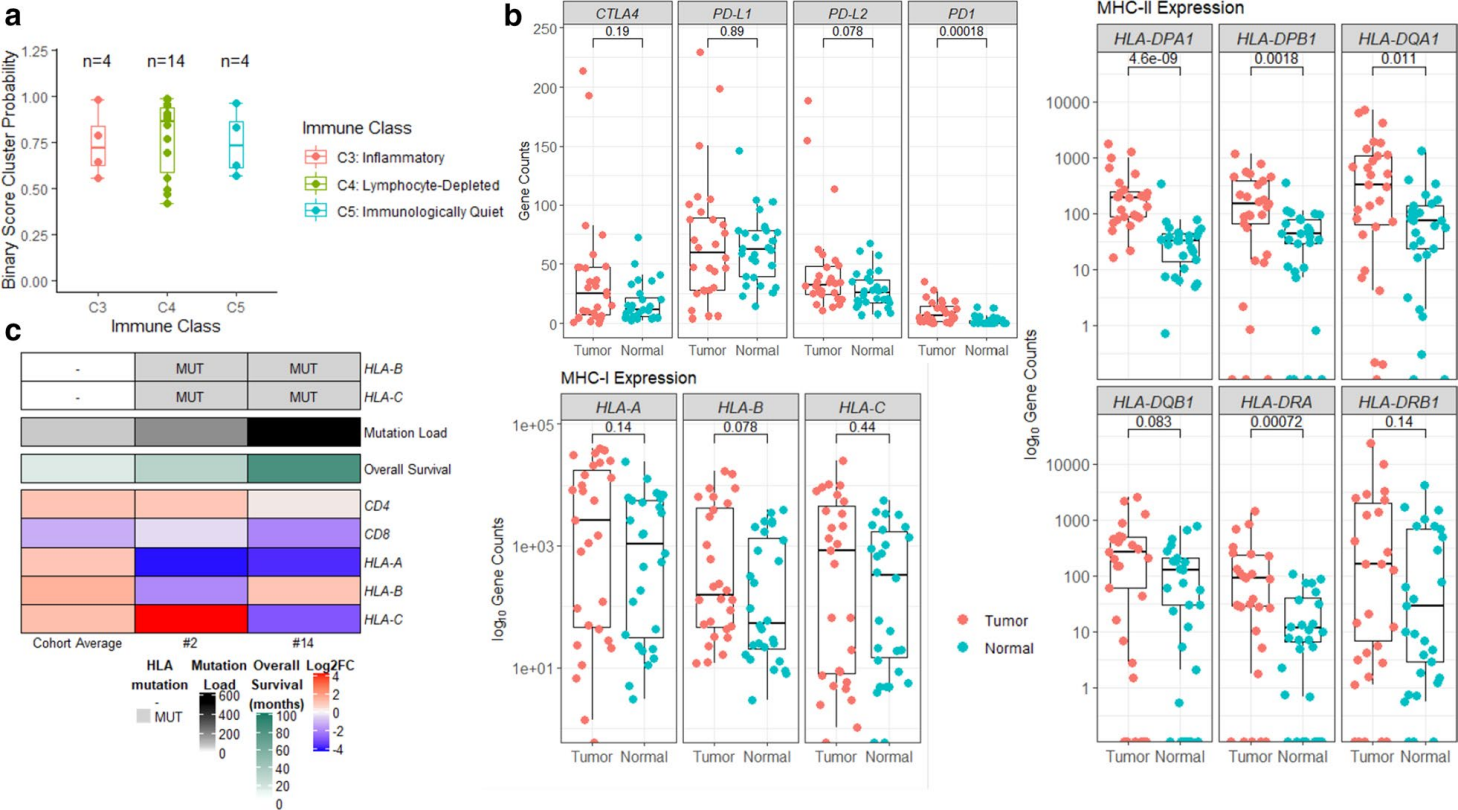
Immunological profiling and immunomodulatory gene expression. **a** Distribution of immune subtypes among tumors in our cohort, defined by RNA sequencing immunologic gene expression analysis following the classification scheme described by Thorsson et al. [69] and using a binary cluster probability threshold of at least 0.4 for sufficiently valid classification. Each dot represents the clustering probability (y-axis), colored by immune class (inflammatory or “C3” [red], lymphocyte-depleted or “C4” [green], immunologically quiet or “C5” [blue]). **b** Expression of immunomodulatory genes: (log_10_) normalized gene counts are plot for each tumor and normal, differential expression are tested by Wilcox Rank Sum test and *p* value reported. **c** Profile of overall tumor mutation load and expression of immunomodulatory genes in two patients (#2 and 14) with co-occurring *HLA-B* and *HLA-C* mutations, compared to (and color-coded against) the cohort average (n = 28)

Immunologic gene expression analysis using RNA sequencing was performed on all tumors, with resultant classification into immune subtypes using previously reported methodology from The Cancer Genome Atlas [69]. A binary cluster probability of at least 0.4 was applied as a threshold for valid classification, with 6 tumors in our cohort omitted due to insufficient probability scores for subgrouping. The majority of evaluable tumors in our cohort had immune signatures consistent with lymphocyte-depleted (“C4”, n = 14 [50%]) or immunologically quiet (“C5”; n = 4 [14%]) subtypes (Figs. 1 and 6a). Four (14%) tumors were classified by immunogenic analysis as having an inflammatory (“C3”) subtype, including the longest survivor in our cohort (Patient #14 [overall survival: 82 months]). There was a trend toward improved overall survival among patients whose tumors were consistent with this inflammatory subtype, compared to those with lymphocyte-depleted or immunologically quiet subtypes (median: 18.5 vs 11.5 months; *p* = 0.14). Although no significant correlation between immune subtypes and post-RT imaging response was identified, three of four patients with PD after radiotherapy had tumors characterized by the lymphocyte-depleted subtype, whereas the three of four patients whose tumors were characterized by the inflammatory subtype exhibited a favorable (non-PD) response to irradiation. Immune subtype (inflammatory vs lymphocyte-depleted or immunologically quiet) did not correlate with histone mutation status (H3.3 vs H3.1; *p* = 0.86). No association between steroid (dexamethasone) use within 3 months of death and immune subtype at autopsy was observed.

#### Expression of immunomodulatory genes

RNA sequencing analyses revealed overexpression of programmed cell death-1 (*PD1*) among the tumors in our cohort compared to normal brain tissue (with no significant correlation with post-RT radiographic response group), suggesting consistent T-lymphocyte infiltration (Fig. 6b). Major histocompatibility complex (MHC) class II and its lymphocyte binding partner *CD4* were both also overexpressed in tumor compared to normal brain tissue. Expression of MHC class I was similar between tumor and normal brain tissue, and expression of its binding partner *CD8* was correspondingly low. Programmed death-ligand 1 (*PD-L1*) and cytotoxic T-lymphocyte associated protein 4 (*CTLA4*) were similarly expressed between tumor and normal brain tissue (i.e., not overexpressed in tumor). A trend toward higher programmed death-ligand 2 (*PD-L2*) expression in tumor samples was observed, though this did not reach statistical significance (*p* = 0.078). Greater than two-fold increase in gene expression between tumor and normal tissue was observed in 15 (54%) patients for *PD1*, 3 patients (11%) for *PD-L1*, 6 patients (21%) for *PD-L2*, and 11 (39%) patients for *CTLA4*. When comparing across tumors within the three immune subgroups (C3, C4, C5), there was no significant difference in tumor expression of *PD1*, *PD-L1*, *PD-L2*, or *CTLA4* or in the proportion of tumors exhibiting greater than two-fold increase in expression compared to normal of any of these genes.

#### HLA mutations and mutation load

Given reports of greater mutational burden and corresponding neoantigen levels among tumors with somatic HLA gene mutations [11], we performed a subanalysis evaluating mutation load among the aforementioned four patients with *HLA-B* and/or *HLA-C*-mutant tumors, compared with the rest of the cohort. All four patients had greater than the cohort-average number of mutations, and the two patients whose tumors harbored co-occurring *HLA-B* and *HLA-C* mutations (Patient# 2 and 14) had significantly higher number of mutations (n = 554 and 193, compared to cohort average of 83; Fig. 6c). Additionally, among patients whose tumors harbored more than the cohort average number of mutations, all but one were either in the SD or PR post-RT imaging response groups.

## Discussion

To our knowledge, this is one of the first studies to apply a radiogenomics approach to a cohort of patients with DIPG in order to (1) investigate relationships between MR imaging phenotypes at post-RT time points (focused on disease response) with tumor molecular profiles identified through extensive genome-wide sequencing analyses, and (2) further explore the radiographic, clinical, and biological heterogeneity of this disease.

Utilizing MR imaging within 2 months of completion of irradiation, patients were divided into three radiographic disease response groups (PR, SD, PD) based on established RANO and RAPNO criteria [13, 14]. Although no difference in prognosis was observed among patients across the three radiotherapy response groups, patients with PD had worse overall survival compared to patients without PD (PR or SD combined), irrespective of age or concurrent radiosensitizer use. As an association between response to irradiation and survival outcomes in DIPG has not been consistently demonstrated [9, 22, 28, 29], though the favorable prognostic impact of tumor reduction following radiotherapy was observed in at least one study [61], our findings deserve further investigation within a larger patient cohort, but provide important preliminary insight into clinically distinct subgroups of DIPG with potential corresponding radiogenomic differences, as described below.

Notably, there was a trend toward different proportions of *H3F3A* (H3.3) and *HIST1H3B* (H3.1) mutations among patients across the three post-irradiation imaging groups, with *H3F3A* mutations unanimous among patients with PD (100%) and *HIST1H3B* mutations more common among patients with PR (67%). These results are consistent with prior work by Castel et al., demonstrating poorer response to radiotherapy among DIPG tumors harboring *H3F3A* mutations compared to *HIST1H3B* mutations [10], and expand on recent radiogenomics research by Jaimes et al., identifying differences in diagnostic MRI characteristics (ADH histogram parameters) among *H3F3A*- and *HIST1H3B-*mutated tumors [37]. No statistical difference in overall survival was observed between patients whose tumors were characterized by *H3F3A* versus *HIST1H3B* mutations in our cohort. This finding, though similar to results recently published by Leach et al. [45], is in contrast to reports of worse overall survival among *H3F3A*-mutant tumors by Castel et al., Hoffman et al., and Jaimes et al. (trend observed in the latter study, though did not reach statistical significance) [10, 33, 37] and therefore should be interpreted cautiously in light of the small sample size. Nonetheless, the aforementioned findings collectively suggest that histone mutation status, though not uniformly prognostic of overall survival in the published literature, may help define biologically unique subtypes of DIPG with different diagnostic and post-RT imaging features, and aid in predicting patients’ clinical trajectories.

Differential gene expression of pathways with oncologic significance was observed between tumors in the three imaging response groups. Epithelial-mesenchymal transition (EMT) has been proposed as an important, likely upregulated, element underlying the diffuse, invasive biology of DIPG [51] and correspondingly, genes associated with this pathway were consistently overexpressed in tumors in our cohort, compared to normal brain tissue. However, patients with PD on post-RT MRI exhibited further enrichment of EMT-related genes, in comparison to patients with either PR or SD, irrespective of histone mutational status, suggesting that the poorer radiographic response to irradiation and/or worse overall survival in the former may be in part related to tumor invasiveness and metastatic potential. Our findings of increased cortical metastases at autopsy among patients with PD post-RT compared with patients with PR or SD further support this. Additionally, whereas the above differential gene expression findings did not appear dependent on histone mutational status, *H3F3A*-mutant tumors in our cohort were significantly more likely to have distant tumor spread to the upper spinal cord at autopsy than *HIST1H3B*-mutant tumors, in agreement with Castel et al., who observed increased metastatic recurrences and gene enrichment of metastasis-related pathways among patients with *H3F3A* mutations [10]. Our results, together with those of Castel et al., suggest that upregulation of genes related to EMT and histopathologically-confirmed distant tumor spread at autopsy are observed in a subset of DIPG patients who may exhibit poorer radiographic response to radiotherapy and/or higher likelihood of harboring *H3F3A* mutations. Further research is necessary to improve early identification of these patients at risk of disseminated disease who may benefit from upfront craniospinal irradiation (CSI). Although the use of CSI has not been extensively studied in this population, there is emerging evidence to suggest feasibility and potential benefit of CSI in patients with metastatic DIPG and other high-grade gliomas at diagnosis or progression that deserves additional exploration [31, 53, 57].

Downregulation of genes involved in oxidative phosphorylation and calcium signaling pathways was consistently demonstrated in all DIPG tumors in our cohort, compared to normal brain tissue, without significant differences across radiographic response groups. These findings are in agreement with research by Shen et al. demonstrating that pediatric high-grade gliomas undergo metabolic reprogramming, favoring glycolysis over oxidative phosphorylation, and therefore shifting glucose metabolism to mitochondrial oxidation represents a potential therapeutic target [67]. Additionally, our results are consistent with prior work by Deng et al. suggesting downregulated genes related to calcium signaling may be a key mechanism in the development and progression of DIPG [18], and potentially targetable via a combination of calcineurin and receptor tyrosine kinase/PI3K pathway inhibition [70].

Whereas a trend toward higher proportion of *TP53* and *ACVR1* aberrations in *H3F3A*- and *HIST1H3B*-mutant tumors, respectively, was identified, consistent with prior reports [10, 40] and a trend toward higher proportion of *PIK3CA* mutations in patients with PR versus non-PR (PD or SD) was observed, there was no significant difference in the presence of these or other known, non-histone, clinically relevant genetic alterations across patients in the three imaging response groups. Analyses may be limited by the small sample size and should be replicated on a larger scale in future studies, especially given recent evidence suggesting a potential role of *TP53* mutations in driving resistance to radiotherapy in DIPG [73]. However, genome-wide sequencing revealed the presence of *DYNC1LI1* mutations in three patients, who all exhibited PR to radiotherapy, whereas this alteration was not detected in any of the patients with SD or PD (and did not correlate with immune subgroup classification, which also appeared to have prognostic relevance [discussed further below]). *DYNC1LI1* encodes part of a protein complex (dynein cytoplasmic 1 light intermediate chain 1) responsible for converting energy from ATP hydrolysis into mitotic spindle function, with proposed important roles in cell division and migration [59, 68]. Although not previously reported in DIPG to our knowledge, altered expression of *DYNC1LI1* has been described in other malignancies (colorectal cancer and breast cancer in adults), with potential impact on therapy sensitivity [5, 12, 34]. Additionally, the presence of alterations in *SRGAP3* and *OR7E24* genes, which have also not previously been described in DIPG, were identified in a subset of patients and associated with overall survival (worse and better, respectively). *SRGAP3* has been implicated as a potential oncogenic driver in low-grade gliomas, when fused with *RAF1*, resulting in constitutive activation of the ERK/MAPK pathway [21, 44], and altered expression of the SRGAP protein has been demonstrated in breast cancer [41]. All five patients in our cohort with *SRGAP3-*mutant tumors were characterized by an amino acid change at the identical position (1081) reported in colorectal cancer [24]. Aberrations and subsequent abnormal expression of *OR7E24* have been observed in glioblastoma, hepatocellular carcinoma, and stomach cancer [15, 77], with an association with microsatellite instability and high tumor mutational burden in the latter. As the functional consequences of the aforementioned genetic alterations of *DYNC1LI1*, *SRGAP3*, and *OR7E24* in DIPG are unknown (and the three patients with *DYNC1LI1*-mutant tumors had distinct mutations), these findings should be interpreted cautiously and further conclusions are limited; however, future investigation into the role that *DYNC1LI1*, *SRGAP3*, and *OR7E24* aberrations may play in DIPG, in regard to potential tumorigenesis, response to irradiation, and/or prognosis, will be critical.

An adequate understanding of the immunologic profile of DIPG is critically important, especially given ongoing investigation into potential immunotherapeutic targets, including current clinical trials studying anti-PD-1 monoclonal antibodies (NCT03130959, NCT02359565), as well as emerging pre-clinical evidence suggesting efficacy of anti-GD2 chimeric antigen receptor (CAR) T cells in orthotopic xenograft models of this disease [2, 52]. To our knowledge, our study is the first to utilize bulk RNA sequencing data to immunologically classify DIPG tumors, applying an immuno-oncology multi-omics analytic approach established by Thorsson et al. [69]. The majority of tumors in our cohort had RNA sequencing profiles consistent with the lymphocyte-depleted (“C4”) or immunologically quiet (“C5”) immune subtypes. These findings align with the non-inflammatory tumor microenvironment that has previously been described in DIPG by Lin et al. and Lieberman et al., with most DIPG tumors (both pre-treatment biopsy and post-treatment autopsy specimens) characterized by low numbers of infiltrating CD3 + T lymphocytes and decreased expression of chemokines and cytokines [47, 48]. However, four tumors in our cohort were classified as having an inflammatory (“C3”) subtype, and a trend towards improved overall survival was observed among these patients (including the longest overall survivor) compared with patients whose tumor profiles were consistent with the lymphocyte-depleted or immunologically quiet subtypes, suggesting that tumors with an inflammatory microenvironment may have a better prognosis. Similarly, although interpretation is limited by the small sample size, a possible association between immune subtype and post-RT imaging was observed, with more favorable (non-PD) radiographic response to irradiation occurring among most patients whose tumors were characterized by the inflammatory subtype, whereas the tumors of all but one patient with PD were characterized by the lymphocyte-depleted subtype. Furthermore, differential expression of genes involved in inflammation and immune-mediated pathways was identified between patients in the PR versus PD post-RT response groups, and enrichment of inflammatory pathway genes was demonstrated among patients within the SD group with longer subsequent disease stability, though future, larger-scale corroboration will be critical.

Additionally, patients whose tumors harbored mutations in *HLA-B*, *HLA-C,* and/or *OR7E24* exhibited a trend toward improved overall survival (although suspected to be pathogenic, the exact functional consequences of these mutations [i.e., increased or decreased expression] is unknown and limits conclusions). Tumor mutational burden and subsequent neoantigen availability for T-cell recognition is an important indicator of potential response to immunotherapy, with DIPG tumors previously shown to have low mutational load, especially compared to adult glioblastoma counterparts [48]. Recent reports have demonstrated higher overall mutation burden and corresponding greater neoantigen levels in colorectal tumors harboring somatic HLA genetic alterations [11], as well as in gastric adenocarcinoma cancers characterized by *OR7E24* mutations and subsequent microsatellite instability [15]. In our cohort, the aforementioned five patients with *HLA-B*, *HLA-C*, and/or *OR7E24*—mutant tumors all had a higher number of mutations than the group average. In addition, among patients whose tumors harbored greater than the cohort average number of mutations, all but one had PR or SD to radiotherapy. Confirmation of these findings in a larger cohort will be essential, but these collective immunologic profiling results provide preliminary therapeutically-relevant information. Taken together, although the DIPG tumor microenvironment is often immunologically “cold”, there appears to be a radiogenomically distinct subset of patients whose tumors have a more inflammatory profile and/or higher mutational burden, with a trend toward improved overall survival (even in the absence of immunotherapy) and more favorable response to radiotherapy, which appears to be irrespective of histone mutation (H3.3 vs H3.1) status. Given the higher likelihood of immunotherapy efficacy in this subset, further investigation to identify these patients upfront, in addition to assessing post-irradiation imaging, will be critical. Research into preferentially utilizing bevacizumab as a steroid-sparing agent in this subgroup of DIPG patients with a more inflammatory tumor phenotype, in order to preserve immune response and optimize effectiveness of immunotherapy, will also be necessary.

Furthermore, DIPG tumors in our cohort collectively highly expressed MHC class II with correspondingly high CD4 + infiltrating immune cell expression compared to matched normal brain tissue, suggesting intact antigen presentation machinery, yet *PD-L1* and *CTLA4* expression were low, signifying limited primary immune response. However, a trend toward higher expression of *PD-L2* was observed. As *PD-L2* is highly expressed on antigen presenting cells and T lymphocytes, has greater (more than fourfold) affinity to PD-1 compared with PD-L1, and is similarly responsible for blocking T-cell response [43, 64], PD-1 inhibition (as opposed to PD-L1 inhibition) may be a more efficacious immunotherapeutic target in this subset of DIPG patients.

Finally, additional MR imaging features at diagnosis, first post-RT assessment, and at subsequent clinically relevant time points were analyzed for prognostic significance. Peripheral ring enhancement and necrosis at diagnosis (though the latter was present at higher prevalence than previously described) were associated with poorer overall survival in our cohort, in accordance with prior reports [33, 39, 45, 62]. Expanding upon this previous research, which largely focused on diagnostic MRI characteristics [33, 39, 45, 62], as well as adding to recent studies suggesting an unfavorable prognostic impact of increased enhancement and decreased relative cerebral blood volume following radiotherapy [9, 61], we observed worse overall survival among patients whose first post-RT MRI exhibited presence of peripheral ring enhancement, necrosis, and diffusion restriction, as well as new and/or increasing peripheral ring enhancement or necrosis, compared to diagnostic MRI. Most of these features identified on MRI at the time of best response or first radiographic progression similarly conferred a worse prognosis. Additionally, a significant difference in the proportion of patients with new and/or increasing enhancement on post-RT imaging was identified when comparing the three disease response groups (PD > SD > PR); no significant association between other radiographic features and response to radiotherapy was observed, albeit potentially limited by the small sample size. Although it will be essential to corroborate these preliminary findings in larger cohorts, this data provides important, new prognostic implications of post-diagnosis MR imaging features, including worse overall survival associated with present, new, and/or increasing enhancement and necrosis both after irradiation and at first radiographic progression.

Our study has several limitations, including a small sample size, decreasing generalizability of our findings as well as our ability to identify statistically significant associations in some instances or perform multivariate analyses. Further exploration and corroboration of findings in a larger cohort will therefore be critically important and is currently planned using data from the International DIPG/DMG Registry. Additionally, genome-wide sequencing analyses were performed on tumor tissue obtained at autopsy and following treatment, not on diagnostic biopsy specimens due to lack of availability. We therefore cannot rule out the possibility of temporal or therapy-related genomic heterogeneity confounding our results. However, previous reports comparing the molecular profiles of paired DIPG tumors from diagnosis and autopsy demonstrated conservation of H3 K27M mutations and associated key genetic alterations (*TP53*, *ACVR1*, *PIK3CA*, *PDGFRA*) [54, 65]. One of the two above patients with *HLA-B*, *HLA-C,* and *OR7E24* mutations and high overall tumor mutational burden received treatment with temozolomide and re-irradiation, potentially leading to mutagenesis; however, when comparing patients with higher or lower than the cohort average mutational load at autopsy, there was no significant difference in the proportion who underwent prior alkylating chemotherapy and/or re-irradiation. Future research is necessary to improve our understanding of how the genomic profiles and mutational load of DIPG tumors may change over time and following potentially mutagenic treatment to optimize interpretation of molecular findings at autopsy. In addition, expansion of our post-mortem DIPG tumor immunologic profiling analysis with immunohistochemical validation, comparison to pre-treatment biopsy specimens, and correlation with systemic therapy received, including specific details of concurrent steroid use, is essential and currently planned. Lastly, radiographic features were assessed visually by a single reviewer (albeit one with extensive experience evaluating neuro-imaging in patients with DIPG). Although subjective, visual assessment was (1) performed in a standardized fashion utilizing previously established and validated criteria from a recent study of baseline imaging in 357 patients with DIPG [45], (2) informed by bi-dimensional quantitative measures, and (3) remains the standard-of-care in neuro-oncology, allowing results to be directly clinically applicable. However, further evaluation and confirmation of our imaging findings using more detailed radiomic quantitative methods such as volumetrics, quantitative diffusion indices, and texture-indices [25], with correlative genomic analyses, will be critical.

Despite these limitations, our results provide valuable insight into the radiographic, clinical, and biological heterogeneity of DIPG with potential implications for treatment. Utilizing an innovative radiogenomics approach in a cohort of 28 patients with DIPG, we report several therapeutically relevant findings, which deserve corroboration in future, larger-scale study planned with data from the International DIPG/DMG Registry: (1) Certain radiologic features on first and subsequent post-RT MRIs are associated with worse overall survival, including PD (compared with SD or PR) following irradiation as well as present, new, and/or increasing peripheral ring enhancement, necrosis, and diffusion restriction. (2) Upregulation of EMT-related genes and distant tumor spread at autopsy are observed in a subset of DIPG patients who may exhibit poorer radiographic response to irradiation and/or higher likelihood of harboring *H3F3A* mutations, the latter in accordance with results previously published by Castel et al. [10]. Given the potential benefit of upfront craniospinal irradiation in these patients at risk of disseminated disease, further research to improve early identification is essential. (3) Genetic aberrations were identified in certain patient subgroups, such as *DYNC1LI1* mutations in patients with PR on post-RT MRI; future investigation into a potential role in DIPG tumorigenesis and/or treatment sensitivity is necessary. (4) Whereas most DIPG tumors have an immunologically “cold” microenvironment, there appears to be a subset of patients whose tumors harbor a more inflammatory genomic profile and/or higher mutational burden, with a trend toward improved overall survival (even in the absence of immunotherapy) and more favorable radiographic response to irradiation, irrespective of histone mutation status. Early recognition and possible steroid-minimizing approaches (i.e., bevacizumab) in these patients who are more likely to benefit from immunotherapy will be key. Although further exploration in larger cohorts is essential, these findings collectively have revealed radiogenomically distinct subgroups of DIPG with unique clinical trajectories and therapeutic targets, to which future treatment strategies should be tailored.

## Supplementary Information

**Additional file 1: Table S1.** Summary of baseline (diagnostic) MR imaging characteristics of patients in our cohort (n=25).
